# Histopathological Analysis of Nodal Disease After Chemoradiation Reveals Viable Tumor Cells as the most Important Prognostic Factor in Head and Neck Squamous Cell Carcinoma, DOI 10.1007/s12105-023-01557-7

**DOI:** 10.1007/s12105-023-01573-7

**Published:** 2023-08-23

**Authors:** Grégoire B. Morand, Aline Golliez, Niels J. Rupp

**Affiliations:** 1 Department of Pathology and Molecular Pathology, University Hospital Zurich, Zurich, Switzerland; 2 Department of Otorhinolaryngology - Head and Neck Surgery, University Hospital Zurich, Zurich, Switzerland; 3 Department of Otolaryngology - Head and Neck Surgery, Sir Mortimer B. Davis – Jewish General Hospital, McGill University, Montreal, QC Canada; 4 Faculty of Medicine, University of Zurich, Zurich, Switzerland; 5 Department of Otorhinolaryngology, Head and Neck Surgery, Lucerne Cantonal Hospital, Lucerne, Switzerland

**Dear Editor,**

We would like to thank the author of the letter to the editor or their interest and comment regarding our recent publication Golliez et al. in Head and Neck Pathology [1]. We would like to provide following reply to their comments: Firstly, we would like to emphasize, that our data refer to the specific situation of nodal persistence in HNSCC after (chemo)radiation. We agree that the survival curves shown in Fig. 4 (panel C and D) do cross and likely violate the proportional hazard assumption, which would limit the validity of a Cox regression model. However, as indicated in the methods and results section, our Cox regression model was built based on the (binary) variable “viable tumor cells” (shown in Supplementary Fig. 1) and not the variable “viable tumor cells area” (shown in Fig. 4) (which authors Sanjari and Shahraki refer to). As demonstrated in Supplementary Fig. 1, there is a clear separation of the curves (after 12 months of follow-up) and violation of the proportional hazard assumption is therefore very unlikely. As for the interpretation of the *p* values, although they do not provide information about the effect size, they do provide a value for the strength of the evidence against H0 [2]. The “relative importance” of a particular variable is not statistically defined, but strong evidence against the null hypothesis may well be considered by many (along with effect size) to be an important factor. Finally, we do agree with the authors that we did not have a validation cohort, and therefore prediction capability cannot be inferred based on our data. This gives opportunity to further studies with larger cohort to assess the exact dose–response relationship of viable tumor cells (area) during the first 12 months and afterwards.

Sincerely,

Grégoire B. Morand, Aline Golliez and Niels J. Rupp.
